# Molecular Characterization and Geographic Distribution of a Mymonavirus in the Population of *Botrytis cinerea*

**DOI:** 10.3390/v10080432

**Published:** 2018-08-15

**Authors:** Fangmin Hao, Mingde Wu, Guoqing Li

**Affiliations:** 1 The State Key Laboratory of Agricultural Microbiology, Huazhong Agricultural University, Wuhan 430070, China; haofangmin@163.com (F.H.); guoqingli@mail.hzau.edu.cn (G.L.); 2 The Key Laboratory of Plant Pathology of Hubei Province, Huazhong Agricultural University, Wuhan 430070, China

**Keywords:** *Botrytis cinerea*, Botrytis cinerea mymonavirus 1, *Mymonaviridae*

## Abstract

Here, we characterized a negative single-stranded (−ss)RNA mycovirus, Botrytis cinerea mymonavirus 1 (BcMyV1), isolated from the phytopathogenic fungus *Botrytis cinerea*. The genome of BcMyV1 is 7863 nt in length, possessing three open reading frames (ORF1–3). The ORF1 encodes a large polypeptide containing a conserved mononegaviral RNA-dependent RNA polymerase (RdRp) domain showing homology to the protein L of mymonaviruses, whereas the possible functions of the remaining two ORFs are still unknown. The internal cDNA sequence (10-7829) of BcMyV1 was 97.9% identical to the full-length cDNA sequence of Sclerotinia sclerotiorum negative stranded RNA virus 7 (SsNSRV7), a virus-like contig obtained from *Sclerotinia sclerotiorum* metatranscriptomes, indicating BcMyV1 should be a strain of SsNSRV7. Phylogenetic analysis based on RdRp domains showed that BcMyV1 was clustered with the viruses in the family *Mymonaviridae*, suggesting it is a member of *Mymonaviridae*. BcMyV1 may be widely distributed in regions where *B. cinerea* occurs in China and even over the world, although it infected only 0.8% of tested *B. cinerea* strains.

## 1. Introduction

*Botrytis* spp., a group of widespread plant pathogenic fungi, can infect more than 1400 plant species, causing gray mold disease on many economically important crops [1]. Besides being pathogens of many plants, *Botrytis* spp. are also ideal hosts for viruses. Among sequenced Botrytis viruses, most positive single-stranded (+ss)RNA viruses were classified into five families—*Alphaflexiviridae*, *Gammaflexiviridae*, *Hypoviridae*, *Narnaviridae*, and a recent proposed family *Fusariviridae*—while most double-stranded (ds)RNA viruses were assigned into three families—*Endornaviridae*, *Partitiviridae*, and *Totiviridae*—and the genus *Botybirnavirus* [2,3,4,5]. In addition, a few sequenced Botrytis viruses, including Botrytis cinerea RNA virus 1 [6], Botrytis ourmia-like virus [7], and Botrytis cinerea negative-stranded RNA virus 1 (BcNSRV1) [8], remained unclassified.

Compared with +ssRNA and dsRNA viruses, (−ss)RNA viruses are rarely reported in *Botrytis cinerea* as well as in other fungi [1]. Mononegaviruses are a group of nonsegmented (−ss)RNA viruses with the genomes of 8.9–19 kb in length, although there are some exceptions [9]. Most reported mononegaviruses infect invertebrates, vertebrates, and plants, whereas only few have been shown to infect fungi [10,11,12,13]. Mononegaviruses are divided into eight families—*Bornaviridae*, *Filoviridae*, *Paramyxoviridae*, *Rhabdoviridae*, *Pneumoviridae*, *Sunviridae*, *Nyamiviridae*, and *Mymonaviridae* [12]—of which *Mymonaviridae* (genus *Sclerotimonavirus*, type species Sclerotinia sclerotiorum negative-stranded RNA virus 1 (SsNSRV1)) is a newly established viral family that accommodates mononegaviruses infecting fungi in the order *Mononegavirales* [14]. In addition, eight other viruses/virus-like contigs, including soybean leaf-associated negative-stranded RNA viruses 1–4 (SlaNSRV1–4), Sclerotinia sclerotiorum negative stranded viruses 2–4 (SsNSRV2–4), and Fusarium graminearum negative-stranded RNA virus 1 (FgNSRV1), are phylogenetically closer to SsNSRV1, and may also belong to *Mymonaviridae* [15,16,17].

In the present study, we describe the genome of a (−ss)RNA virus infecting the fungus *B. cinerea*, namely Botrytis cinerea mymonavirus 1 (BcMyV1). Genomic and phylogenetic analysis indicates that BcMyV1 was most closely related to Sclerotinia sclerotiorum negative-stranded RNA virus 7 (SsNSRV7) [18] and also showed homology to other fungal mononegaviruses. In addition, we also determined the incidence and geographic distribution of BcMyV1 in the population of *B. cinerea* in China.

## 2. Materials and Methods 

### 2.1. Fungal Strains, Culture Conditions, and Biological Characterization

*B. cinerea* strains Ecan17-2 was originally obtained through single conidium isolation from diseased oilseed rape (*Brassica napus*) stem in Shiyan, Hubei Province, China, and strain B05.10 of *B. cinerea* was used as a control [3]. In addition, 508 *B. cinerea* strains from 40 counties/cities in 11 provinces of China were used for testing the presence of BcMyV1. All strains were stored at 4 °C and working culture was established [19]. The mycelial growth was determined on potato dextrose agar (PDA) in petri dishes [3], while the pathogenicity of *B. cinerea* strains was determined on detached *Nicotiana benthamiana* leaves [3,19,20].

### 2.2. dsRNA Extraction and Purification

dsRNAs from *B. cinerea* mycelia was extracted and purified as described previously [20] and was further confirmed based on resistance to DNase I and S1 nuclease (Promega, Madison, WI, USA). The extracted dsRNA was fractionated by agarose gel (1%, *w*/*v*) electrophoresis and visualized by staining with ethidium bromide (1.5 µg/L) and viewing on a UV transilluminator.

### 2.3. cDNA Cloning and Sequencing

After separation by agarose gel electrophoresis, the dsRNA segment (BcMyV1 replication intermediates, approximately 10 kb in size based on the DNA marker) was gel-purified by using AxyPrepTM DNA Gel Extraction Kit (Axygen Scientific, Inc.; Union City, CA, USA) as described by Wu et al. [21]. The cDNAs of BcMyV1 were produced using a random-primer-mediated PCR amplification protocol [6] and were then sequenced [21]. The terminal sequences of the dsRNA were cloned through ligating the 3*′*-terminus for each strand of the dsRNA with the 5*′*-terminus of the 110A adaptor (Table S1) using T4 RNA ligase (Promega Corporation, 2800 Woods Hollow Road, Madison, WI, USA) at 16 °C for 18 h and were then reverse transcribed using primer RC110A (Table S1). The cDNA strands were then used as template for PCR amplification of the 5*′*- and 3*′*-terminal sequences with the primer RC110A and the corresponding sequence specific primers (Figure S1 and Table S1). Cloning of the 3*′*- or 5*′*-terminal sequences of the dsRNA was performed for three rounds. In addition, the internal region of the BcMyV1 genome was also amplified through RT-PCR with four sequence-specific primer pairs (Figure S1 and Table S1). All these amplicons were detected by agarose gel electrophoresis, gel-purified, and cloned into *Escherichia coli* DH5α and sequenced as previously described [19]. All cDNA sequences were assembled to obtain the full-length cDNA sequence of BcMyV1.

### 2.4. Sequence Analysis

Open reading frames (ORFs) in the full-length cDNA sequences of BcMyV1 in strain Ecan17-2 of *B. cinerea* were deduced using the ORF Finder program on the website of the National Center for Biotechnology Information (NCBI, http://www.ncbi.nlm.nih.gov/gorf/gorf.html). The homologous sequences searching for the full-length cDNA sequences and deduced polypeptides of BcMyV1 were carried out at the NCBI database by using the BlastN and BlastP programs, respectively. CDD database (http://www.ncbi.nlm.nih.gov/Structure/cdd/wrpsb.cgi) searching predicted the domains present in the polypeptide sequence. Multiple alignments of the sequences of mononegaviral RNA-dependent RNA polymerase (RdRp) domains in the polypeptides encoded by BcMyV1 and other mononegaviruses were performed using the ClustalW program in MEGA 7.0 [22]. Phylogenetic trees based on the sequences of RdRp domains were constructed using the neighbor-joining (NJ) method and tested with a bootstrap of 1000 replicates to ascertain the reliability of a given branch pattern in MEGA 7.0. Putative transmembrane helices sequences were predicted using the TMHMM server version 2.0 (http://www.cbs.dtu.dk/services/TMHMM/) [23].

### 2.5. Detection of BcMyV1 in *B. cinerea* Population

The total RNAs of 508 *B. cinerea* strains were extracted using the TRIzol^®^ reagent (Invitrogen Corp, Carlsbad, CA, USA) as described previously [19], and the presence of BcMyV1 was determined by using RT-PCR with primer pairs M-RT-F/R (Table S1), which was designed to amplify a specific band of 728 bp in size.

## 3. Results

### 3.1. B. cinerea Strain Ecan17-2 Exhibits Hypovirulence Traits

**Figure 1:**
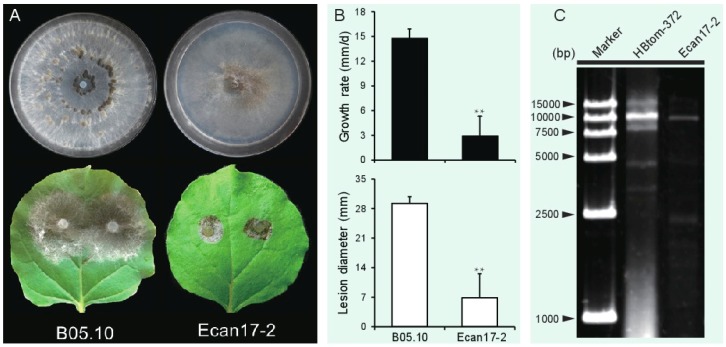
(**A**) Culture morphology (upper, 20 °C, 9 days) and pathogenicity assay (lower, 20 °C, 3 days) of *Botrytis cinerea* strains Ecan17-2 and B05.10 on potato dextrose agar (PDA) and detached *N. benthamiana* leaves, respectively. (**B**) Radial mycelial growth rate (20 °C, upper) on PDA and lesion diameter (20 °C, 72 h, lower) on detached *N. benthamiana* leaves of strains Ecan17-2 and B05.10. “**” indicates a significant difference (*p* < 0.01) between strains Ecan17-2 and B05.10 in both pathogenicity and radial mycelial growth rate. (**C**) Agarose gel electrophoresis of dsRNAs extracted from the mycelia of *B. cinerea* strains Ecan17-2 and HBtom-372.

After cultivation on a PDA plate for 9 days, strain Ecan17-2 formed colonies with no production of sclerotia, whereas strain B05.10 produced massive sclerotia on the colony (Figure 1A). In addition, the radial mycelial growth of Ecan17-2 on PDA, averaging 2.9 mm/day, was significantly slower than that of strain B05.10 (14.8 mm/day). The virulence assay on detached *N. benthamiana* leaves revealed that the average lesion diameter (6.9 mm) caused by strain Ecan17-2 was significantly smaller than that (29.3 mm) of strain B05.10 (Figure 1B).

### 3.2. Genome Analysis of BcMyV1

**Figure 2:**
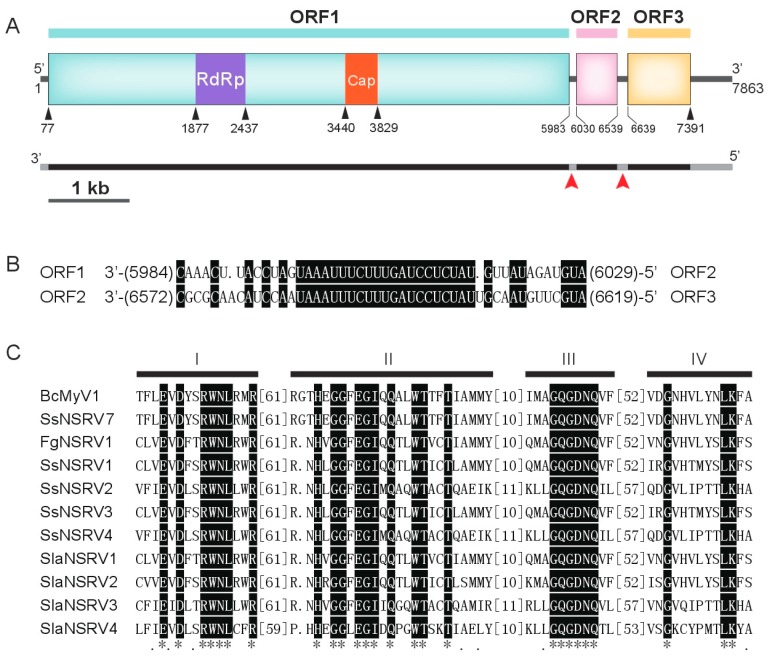
(**A**) Schematic diagram of the genome organization of Botrytis cinerea mymonavirus 1 (BcMyV1). The coding strand of BcMyV1 is 7863 nt long and contains three Open reading frames (ORFs), and the ORF1 encode a protein L of 1968 amino acids (aa), possessing a mononegaviral RNA-dependent RNA polymerase (RdRp) domain and a mRNA-capping region V (Cap) domain. ORF2 and ORF3 encode two proteins of 169 aa and 250 aa, respectively. The black bars indicate the coding regions, and the gray bars represent the untranslated regions (UTRs) on the genome of BcMyV1. Two red arrowheads point out the positions of a 21 nt repeat region on the two UTRs, and the detailed sequence information are listed in (**B**). The numbers in the parentheses indicate the nt positions nearby the parentheses. (**C**) Multiple alignment of the amino acid sequences of RdRp in the protein L encoded by BcMyV1 and other (−ss)RNA viruses. “*” indicates identical amino acid residues; and “.” indicate low chemically similar amino acid residues. The abbreviations of virus names are listed in Table 1.

After DNase I and S1 nuclease digestion, a major dsRNA segment was detected through electrophoresis in the mycelium of *B. cinerea* strain Ecan17-2 with the size of approximately 10.0 kb, which was slightly smaller than the dsRNA-B (Botrytis cinerea fusarivirus 1 (BcFV1), 8411 bp) detected in strain HBtom-372 [3]. The coding strand (GenBank accession no. MH648611) of BcMyV1 was 7863 nt long, with a GC content of 41.6%, possessing three ORFs (ORF1–3) and two short untranslated regions (UTRs) of 76 nt and 472 nt in length at the 5′- and 3′-terminus, respectively. The ORF1 was predicted to encode a putative large polypeptide of 1968 amino acid (aa) residues (Figure 2A), which contains a putative mononegaviral RdRp domain and a mononegaviral mRNA-capping region V (Figure 2A). The ORF2 and ORF3 encoded two proteins of 169 aa and 250 aa in length, respectively. In addition, the 21 nt long repeated sequence 3′-UAAAUUUCUUUGAUCCUCUAU-5′ was detected in the two UTRs between the three ORFs (Figure 2B).

The results of the Blast search showed that the nucleotide sequence of the internal region (10-7829) of the BcMyV1 genome was 97.9% identical to the full-length nucleotide sequence of a contig obtained from metatranscriptomes of *Sclerotinia sclerotiorum* isolates, SsNSRV7 [18]. In addition, the polypeptide encoded by BcMyV1 ORF1 was almost 99.9% identical to the protein L of SsNSRV7 and also showed homology to the protein L of SsNSRV1 (33.1% aa identity), FgNSRV1 (33.8% aa identity), and several (−ss)RNA viruses identified through deep sequencing (Table 1). Four conserved motifs, I–IV, found in mononegaviruses were also identified in the protein L encoded by BcMyV1 ORF1 (Figure 2C). Unlike SsNSRV1, transmembrane (TM) domains (Figure S2) were found at the C-proximal protein L of BcMyV1. However, the proteins encoded by ORF2 and ORF3 showed no significant sequence similarity with proteins in the database of NCBI by using BlastP search.

### 3.3. Phylogenetic Analysis of BcMyV1

**Figure 3:**
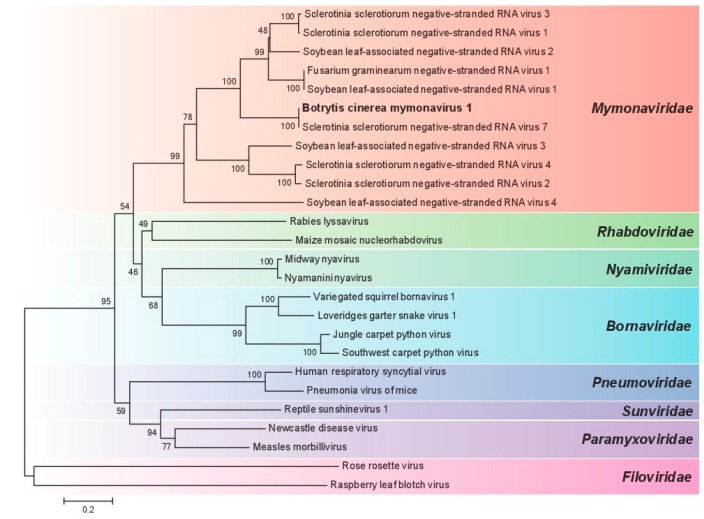
Phylogenetic analysis of Botrytis cinerea mymonavirus 1 (BcMyV1) based on RdRp domain from strain Ecan17-2 of *B. cinerea*.

To define the phylogenetic relationship of BcMyV1 with other viruses in *Mononegavirales* (Table 1), a phylogenetic tree was established based on the mononegaviral RdRp domain. BcMyV1 firstly formed a tight cluster with SsNSRV7 and then clustered with (−ss)RNA mycoviruses from *S. sclerotiorum*, *F. graminearum*, and other viral-like contigs, forming an independent clade of *Mymonaviridae* with the bootstrap support of 99%. In addition, other viruses from *Bornaviridae*, *Sunviridae*, *Filoviridae*, *Rhabdoviridae*, *Paramyxoviridae*, *Pneumoviridae*, and *Nyamiviridae* also formed the corresponding viral family clades (Figure 3). Therefore, we suppose that BcMyV1 should be a member in the virial family *Mymonaviridae*.

### 3.4. Incidence and Distribution of BcMyV1

**Figure 4:**
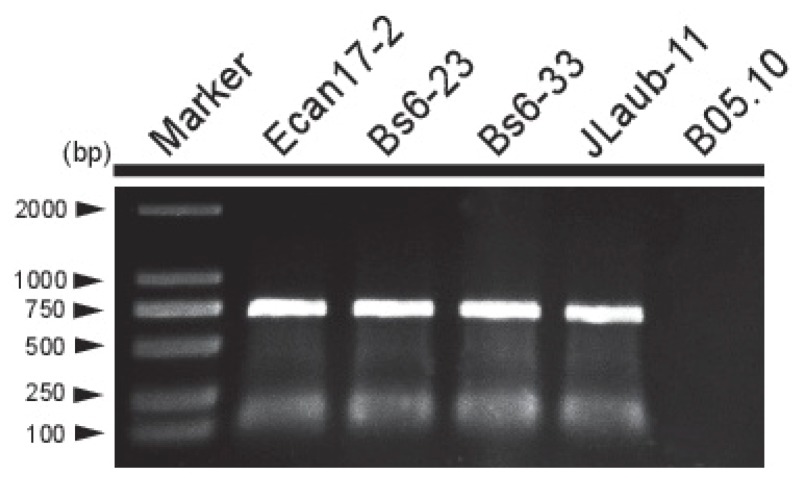
RT-PCR detection of Botrytis cinerea mymonavirus 1 (BcMyV1) in five *B. cinerea* strains. Strains Ecan17-2 and B05.10 served as positive and negative controls, respectively.

**Figure 5:**
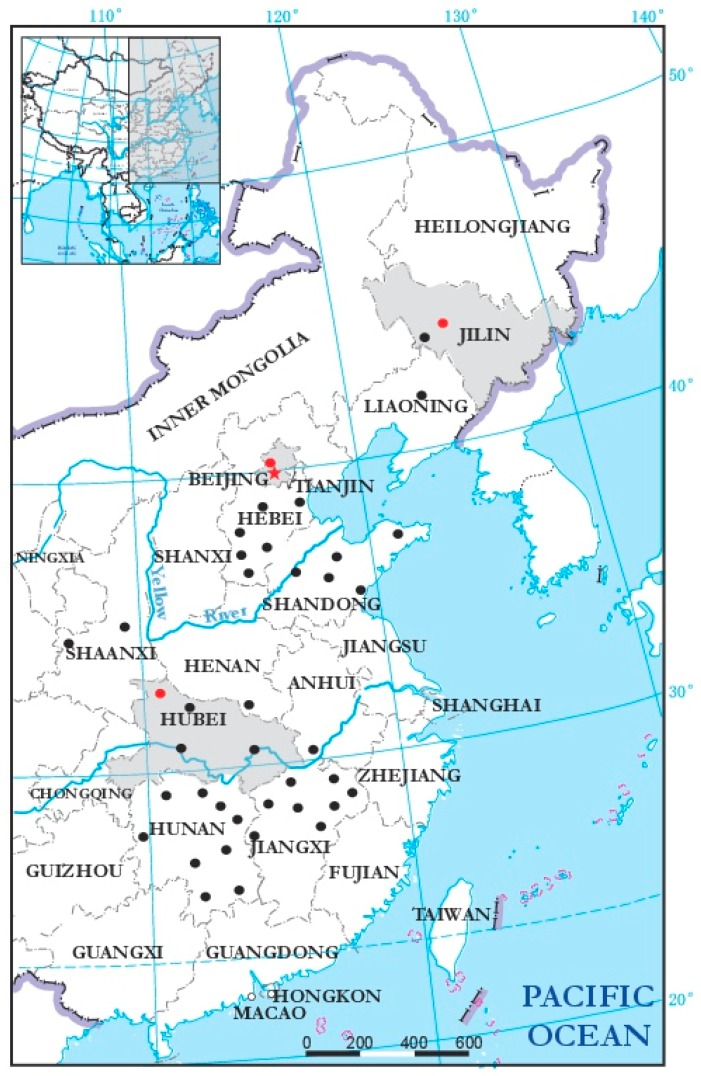
Geographic distribution of Botrytis cinerea mymonavirus 1 (BcMyV1) in 11 provinces of China. The black dots indicate the places where BcMyV1 was not detected, whereas places where BcMyV1 was detected are indicated as red dots and the corresponding provinces are also highlighted in grey on the map.

In order to investigate the incidence and distribution of BcMyV1 in China, 508 *B. cinerea* strains from China were tested for the presence of BcMyV1 by using RT-PCR with the primer pair M-RT-F/R (Table S1). BcMyV1 infection was detected in only 4 out of the 508 (0.8%) tested *B. cinerea* strains (Figure 4 and Figure 5.). In these BcMyV1-infected strains, Bs6-23 and Bs6-33 were collected from the same location (Beijing, China), whereas strain JLaub-11 was collected from Changchun of Jilin Province.

## 4. Discussion

In the present study, we characterized the genome of a (−ss)RNA mycovirus, namely, BcMyV1, infecting the hypovirulent strain Ecan17-2 of *B. cinerea*. Notwithstanding that numerous mycoviruses have been reported in *B. cinerea*, only one case of a (−ss)RNA virus (BcNSRV1) had been characterized [8]. BcNSRV1 is phylogenetically related to members of the genus *Emaravirus* in the viral family *Bunyaviridae*. However, phylogenetic analysis based on RdRp domain indicated that BcMyV1 should belong to the viral family *Mymonaviridae* in the order *Mononegavirales*. As high sequence similarity was observed between BcMyV1 and SsNSRV7, BcMyV1 should be a strain of SsNSRV7 [18]. Nonetheless, the nucleotide sequence of BcMyV1 was longer at both 5′- and 3′-termini than those of SsNSRV7, indicating the sequence of SsNSRV7 in the NCBI database might be incomplete.

In *S. sclerotiorum*, SsNSRV1 infection was closely related to the debilitation symptoms of the infected *S. sclerotiorum* strain, including slow growth on PDA, loss of the ability to produce sclerotia, and pathogenicity on oilseed rape [14]. Similarly, *B. cinerea* strain Ecan17-2 carrying BcMyV1 also displayed reduced mycelial growth on PDA and attenuated virulence on *N. benthamiana*, indicating possible negative effects of BcMyV1 on its host. However, a faint dsRNA segment of approximately 2.4 kb in length (Figure 1C) was also detected in strain Ecan17-2, indicating coinfection of other viruses or defective/satellite RNAs with BcMyV1 [3]. Thus, sequencing the 2.4 kb dsRNA in the traditional way is warranted to ascertain the causal agent of hypovirulence in *B. cinerea* strain Ecan17-2. Moreover, deep sequencing [8] may also be an option to determine the full view of the viral infection in strain Ecan17-2. Generally, two aspects of approach have been used for construction of isogenic lines that aim to elucidate the effects of mycoviral infection on their hosts. Firstly, viruses could be introduced into virus-free strains by using the techniques like pairing-culture [3], virion transfection [21], and construction of infectious cDNA clones [24]. On the other hand, some investigations, including sequential hyphal tip isolation [25], protoplasts/small mycelial fragments regeneration [26], treatment of cycloheximide [27,28], or cAMP-rifamycin [29], and single spore isolation [20] were also explored to cure the viruses in their original strains. Therefore, similar experiments will also be carried out to elucidate the role of BcMyV1 on *B. cinerea* biology in the future.

It is of interest that BcMyV1 was detected in two different fungal species, *B. cinerea* and *S. sclerotiorum*. Although the same mycovirus is rarely detected in different fungi, there are still a few exceptions. In addition to the present case, another mycovirus, Botrytis porri botybirnavirus 1 (BpBV1), was also detected in both *B. porri* and *S. sclerotiorum* [18,21]. We suppose that viral interspecific transmission may frequently occur between *B. cinerea* and *S. sclerotiorum*, although they belong to different genera. Some factors may increase the possibility of viral transmission between the two species. Firstly, *B. cinerea* is a close relative of *S. sclerotiorum*, and their genes share 83% aa identity on average between the two fungi [30]. Thus, viruses may adapt a new host more easily when viral transmission occurs from one host to the other. Secondly, both fungi have a broad host range [1,31], and many plant species are hosts for both *B. cinerea* and *S. sclerotiorum*. Therefore, the contact between the two fungi may frequently occur in small niches under field conditions [30]. Thirdly, viral interspecies transmission through anastomosis has also been reported in a few cases, including from *Aspergillus niger* to *A. nidulans* [32], from *S. sclerotiorum* to *S. minor* [33], and from *Cryphonectria parasitica* to *C. nitschkei* [34], suggesting that viral interspecies transmission by anastomosis between *B. cinerea* and *S. sclerotiorum* might also be possible. Finally, recent studies have shown that insects and mites are also potential vectors during mycoviral transmission [35,36], and similar mechanisms may increase the possible viral transmission between *B. cinerea* and *S. sclerotiorum* as well.

Although –ssRNA viruses have been reported in several fungal species [8,14,15,16,17,18], the information of their incidence and distribution remain unclear. Moreover, investigation of other BcMyV1-infected *B. cinerea* strains may also help us to uncover their effects on *B. cinerea*. On the contrary, unlike strain Ecan17-2, two strains of *B. cinerea* (Bs6-33 and JLaub-11) carrying BcMyV1 grew quickly and produced massive sclerotia, and only one strain, Bs6-23, showed similar culture morphology to that of strain Ecan17-2 (data not shown). This suggests that more complex interactions may exist between BcMyV1 and the *B. cinerea* population. Therefore, genome-wide association study [37,38] may be helpful to further elucidate the role of genetic variation in the response of *B. cinerea* to viral infection. Compared with other viruses infecting *B. cinerea*, including Botrytis cinerea endornavirus 1 [2], Botrytis cinerea hypovirus 1, BcFV1 [3], Botrytis cinerea mitovirus 1 [39], Botrytis virus F, and Botrytis virus X [5], the incidence of BcMyV1 in the Chinese *B. cinerea* population was very low, accounting for only 0.8% in the tested strains. The linear distance between Beijing and Shiyan is over 900 km, and Changchun is over 800 km away from Beijing. These results indicate that BcMyV1 might be widely distributed in regions where *B. cinerea* occurs of China, although the infection rate is low. This may be one reason why (−ss)RNA viruses were rarely reported in the population of *B. cinerea*. Despite the low incidence, BcMyV1 still had a wide geographic distribution and may not be only limited to China, as the homologous virus-like contig was obtained from *S. sclerotiorum* strains in Australia. This suggests BcMyV1 may have a global distribution.

## Figures and Tables

**Table 1:** Percentage of sequence identities between Botrytis cinerea mymonavirus 1 and other mononegaviruses according to the multiple alignments of the full-length protein L and the RNA-dependent RNA polymerase domain.

Family	Virus	Acronym	aa Identity (%)		Accession no.
			Full Sequence	RdRp	
*Mymonaviridae*	Sclerotinia sclerotiorum negative-stranded RNA virus 7	SsNSRV7	99.85	100	MF444285
	Sclerotinia sclerotiorum negative-stranded RNA virus 1	SsNSRV1	33.12	56.68	NC_025383.1
	Sclerotinia sclerotiorum negative-stranded RNA virus 2	SsNSRV2	22.28	41.45	KP900931.1
	Sclerotinia sclerotiorum negative-stranded RNA virus 3	SsNSRV3	33.57	56.15	NC_026732.1
	Sclerotinia sclerotiorum negative-stranded RNA virus 4	SsNSRV4	21.67	39.38	KP900930.1
	Soybean leaf-associated negative-stranded RNA virus 1	SlaNSRV1	32.75	59.36	KT598225.1
	Soybean leaf-associated negative-stranded RNA virus 2	SlaNSRV2	33.12	61.5	KT598227.1
	Soybean leaf-associated negative-stranded RNA virus 3	SlaNSRV3	21.43	37.31	KT598228.1
	Soybean leaf-associated negative-stranded RNA virus 4	SlaNSRV4	17.82	34.57	KT598229.1
	Fusarium graminearum negative-stranded RNA virus 1	FgNSRV1	32.75	59.36	MF276904.1
*Bornaviridae*	Jungle carpet python virus	JCPV	14.84	24.6	MF135780
	Southwest carpet python virus	SWCPV	14.33	21.93	MF135781
	Loveridge’s garter snake virus 1	LGSV1	14.28	23.53	KM114265
	Variegated squirrel bornavirus 1	VSBV1	14.16	25.13	LN713681
*Rhabdoviridae*	Rabies virus	RabV	14.97	23.62	AB517659
	Maize mosaic virus	MMV	15.19	23.12	NC_005975.1
*Paramyxoviridae*	Newcastle disease virus	NDV	14.94	27.69	JF827026.1
	Measles virus	MV	15.44	30.77	NC_001498.1
*Nyamiviridae*	Midway nyavirus	MIDMV	15.81	26.42	NC_012702.1
	Nyamanini nyavirus	NYMV	16.01	26.42	NC_012703.1
*Filoviridae*	Rose rosette virus	RRV	10.57	11.4	HQ871942
	Raspberry leaf blotch virus	RLBV	10.71	10.27	FR823299
*Sunviridae*	Reptile sunshinevirus 1	RSV-1	14.26	24.26	NC_025345
*Pneumoviridae*	Human respiratory syncytial virus	HRSV	13.43	22.68	NC_001781
	Pneumonia virus of mice	PVM	14.22	22.8	NC_006579

## Supplementary Materials

The following are available online at http://www.mdpi.com/1999-4915/10/8/432/s1. Table S1: Oligonucleotide primers/adaptor used in this study. Figure S1: Schematic representation of the strategy used for full cDNA sequence cloning of Botrytis cinerea mymonavirus 1 (BcMyV1). Figure S2: Transmembrane domains prediction for ORF1 encoded polypeptide L of BcMyV1.
