# Supplementary material for: Molecular Characterization and Geographic Distribution of a Mymonavirus in the Population of Botrytis cinerea
Source: Viruses. 2018 Aug 15;10(8):432. doi: 10.3390/v10080432 (PMC6116283; doi:10.3390/v10080432)
Supplement: Supplementary file 1 [file viruses-10-00432-s001.pdf]

Figure S1.

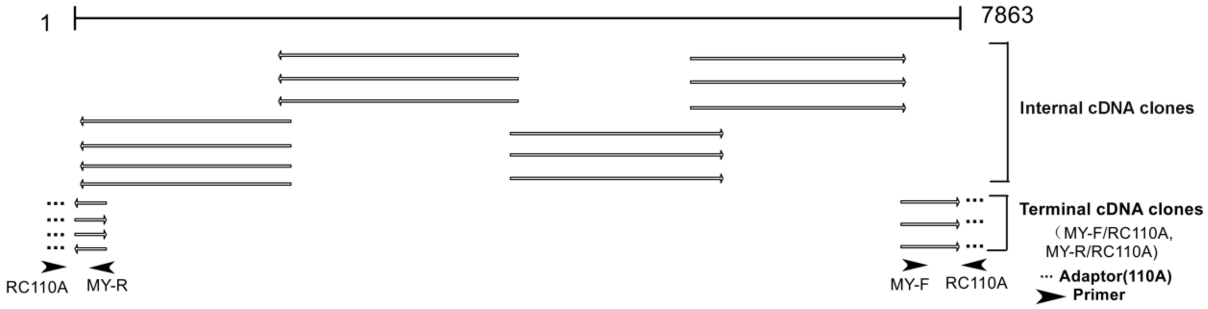

Figure S2.

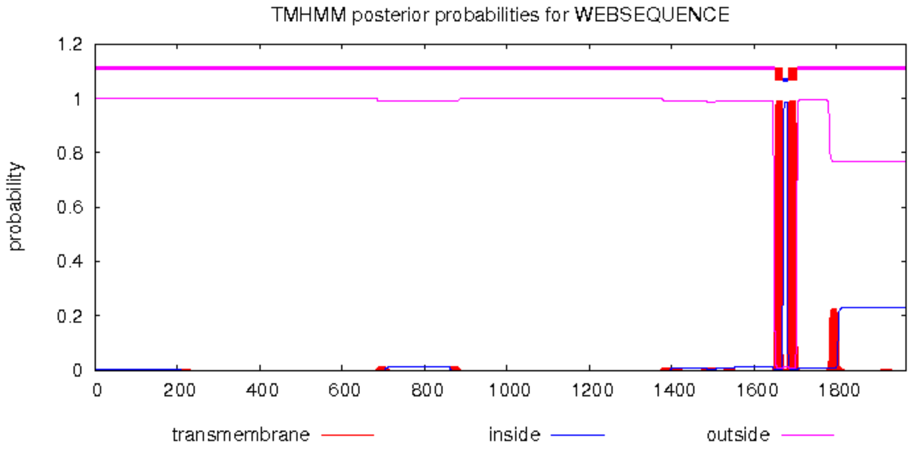

Table S1. Oligonucleotide primers/adaptor used in this study.

Name	Sequence (5′→ 3′)	Position [1]	Polarity [2]
	for cDNA cloning		
MY-1F	ATTGTCTACCCGCGACGTAA	45-64	+
MY-1R	ACGGTGGTGTGCCTCATAC	1914-1932	–
MY-2F	ACGGAGACCAAGCAACGACTC	1820-1840	+
MY-2R	GGAGGATCGCAAACCACGG	3936-3954	–
MY-3F	TAATGCCTCAATAGGCTTCG	3880-3899	+
MY-3R	TCAGGGCGTCATTGCGTAGT	5758-5774	–
MY-4F	CTATGTTCGCTTACGACGCA	5475-5494	+
MY-4R	ATTCGGGACCTTGATCGGTG	7370-7389	–
MY-F	TGAGCATGGAGAGACAGAT	7340-7358	+
MY-R	AACGCGTTTACTCGAGGAGG	278-297	–
110 (3′-adaptor)	TATCTTATCGGCGTGTCCCCC	to 3′-end of dsRNA	+/–
RC110A (primer)	GGGGGACACGCCGATAAGATA	complementary to the adaptor 110A	–/+
	for RT-PCR detection		
M-RT-F	TAATGCCTCAATAGGCTTCG	3880-3899	+
M-RT-R	AGATGCTGCAACCGCACCATGCC	5136-5158	–

[1] Positions for the PCR primers or the adaptor in the cDNA of Botrytis cinerea mymonavirus 1 (BcMyV1) were labeled in Figure S1.

[2] Polarity refers to the positive strand (+) and the negative strand (–) of BcMyV1.
